# The effect of post-discharge educational intervention on patients in achieving objectives in modifiable risk factors six months after discharge following an episode of acute coronary syndrome, (CAM-2 Project): a randomized controlled trial

**DOI:** 10.1186/1477-7525-8-137

**Published:** 2010-11-22

**Authors:** Javier Muñiz, Juan J Gómez-Doblas, María I Santiago-Pérez, Iñaki Lekuona-Goya, Nekane Murga-Eizagaetxebarría, Eduardo de Teresa-Galván ß, José M Cruz-Fernández, Alfonso Castro-Beiras

**Affiliations:** 1 Instituto Universitario de Ciencias de la Salud de la Universidad de A Coruña. Spain; 2 Servicio de Cardiología. Hospital Universitario Virgen de la Victoria, Malaga. Spain; 3 ODDS, S.L., La Coruña. Spain; 4 Servicio de Cardiología. Hospital Galdakao, Vizcaya. Spain; 5 Servicio de Cardiología. Hospital de Basurto, Vizcaya. Spain; 6 Servicio de Cardiología. Hospital Universitario Virgen Macarena, Sevilla. Spain; 7 Servicio de Cardiología. Complejo Hospitalario Universitario de A Coruña, Servicio Galego de Saúde. La Coruña. Spain

## Abstract

**Objectives:**

We investigated whether an intervention mainly consisting of a signed agreement between patient and physician on the objectives to be reached, improves reaching these secondary prevention objectives in modifiable cardiovascular risk factors six-months after discharge following an acute coronary syndrome.

**Background:**

There is room to improve mid-term adherence to clinical guidelines' recommendations in coronary heart disease secondary prevention, specially non-pharmacological ones, often neglected.

**Methods:**

In CAM-2, patients discharged after an acute coronary syndrome were randomly assigned to the intervention or the usual care group. The primary outcome was reaching therapeutic objectives in various secondary prevention variables: smoking, obesity, blood lipids, blood pressure control, exercise and taking of medication.

**Results:**

1757 patients were recruited in 64 hospitals and 1510 (762 in the intervention and 748 in the control group) attended the six-months follow-up visit. After adjustment for potentially important variables, there were, between the intervention and control group, differences in the mean reduction of body mass index (0.5 vs. 0.2; p < 0.001) and waist circumference (1.6 cm vs. 0.6 cm; p = 0.05), proportion of patients who exercise regularly and those with total cholesterol below 175 mg/dl (64.7% vs. 56.5%; p = 0.001). The reported intake of medications was high in both groups for all the drugs considered with no differences except for statins (98.1% vs. 95.9%; p = 0.029).

**Conclusions:**

At least in the short term, lifestyle changes among coronary heart disease patients are achievable by intensifying the responsibility of the patient himself by means of a simple and feasible intervention.

## Background

Clinical practice guidelines recognise a series of pharmacological and hygienic-dietetic measures as being effective for secondary prevention in patients with acute coronary syndrome [1]. However, in spite of the improvement in recent years in measures to assure compliance in secondary prevention in post-infarct patients, there still exists considerable room for improvement [2-9]. Adherence to the treatment recommended has a favourable effect on the evolution of those patients who comply. In patients with coronary heart disease, following the recommendations of clinical guidelines in secondary prevention has a favourable effect on morbimortality in the follow-up period [10-12]. Multiple strategies have been developed to improve the adherence of patients to the recommendations, with more or less impact on the achievement of aims [13,14]. But, on occasion, these are focused exclusively on measures of pharmacological prescription, and in others there is no control group against which to assess the impact of the measures adopted [15].

One last important aspect is the continuity of care after hospital discharge. Measures in this respect have been shown to be more effective the sooner they are applied after the provision of care (such as when the hospital discharge report itself is issued) [16]. Many strategies to improve hospital care and recommendations at discharge are widely known, but rarely do they attempt to go beyond the time of the actual submission of the discharge report [17], even when the effectiveness of carrying out early monitoring of patients discharged after an acute coronary event is acknowledged [18].

The objective of our study is the evaluation after six months of follow-up of a programme of intervention at hospital discharge, easily embeddable in the daily clinical practice, aimed at increasing the proportion of patients that meet objectives in modifiable cardiovascular risk factors among patients who suffered an acute coronary syndrome, and which its main elements are the negotiation between the patient and the physician on the treatment objectives and steps to be taken and a reinforcement visit two months after discharge.

## Methods

Open-label randomized controlled trial performed with 64 Spanish hospitals participating, in which a maximum of 30 patients were recruited at each one, all of whom were discharged consecutively after suffering acute coronary syndrome.

Finally, 1,757 patients were assigned to the Intervention Group (n = 867) or the Control Group (n = 890) by means of stratified randomization by centre and with concealment of allocation sequence. The unit of randomization was the patient and the stratification by centre was done to remove the effect of the hospital by obtaining groups of equal size (intervention and control) in every hospital. These were not selected at random but made up a convenient sample of Spanish hospitals, many of which had already taken part in the research group's previous studies [16]. All participating centers were either public or concerted with the public system. The hospitals covered a wide range of size (measured by the number of beds) and complexity. 16 hospitals had less than 400 beds, 22 between 400 and 800 and 26 hospitals had more than 800 beds. 39 hospitals (61%) had cardiac catheterization laboratory. There were hospitals from all over Spain, with sixteen out of the seventeen autonomous communities of Spain represented with at least one hospital as well as thirtyfive out of the fifty provinces in which Spain is divided.

The study was carried out during 2004 and 2006. The study was approved by the Clinical Ethics and Research Commission of Galicia (CEIC). All patients signed giving their informed consent to participate in the trial.

### Inclusion criteria

Patients of both genders admitted for acute coronary syndrome with or without ST-segment elevation, discharged with a diagnosis of Q-wave or non-Q-wave acute myocardial infarction (AMI) or unstable angina with sufficient cultural level to be able to understand the intervention and the ability to fulfill the schedule of proposed further visits (at two and six months). Patients under 18 or above 80 years of age were not included, as it was the case with those presenting accompanying illnesses which would cause unfavourable prognosis in the following months or which would prevent their participation. All patients received the usual information on discharge.

### Definition of the intervention

In this experimental study the intervention is realized upon the physician/patient relationship. The intervention includes a personalised interview at discharge with patient and nearest next-of-kin, in which physician and patient discuss and sign an agreement with the patient-specific secondary prevention procedures and therapeutic aims. Both physician and patient keep a copy of the signed agreement. Written back-up information is also given, some designed for this project and other based on material available from the Spanish Society of Cardiology, the Spanish Heart Foundation and the American Heart Association ("After Your Heart Attack", "Understanding and Controlling Your Cholesterol ", "The Effects of Smoking ","Warning Signs and Actions in the Face of a Heart Attack" "Get Moving", "Understanding and Controlling Your Blood Pressure", "Controlling Your Weight", among others) (materials available upon request), as well as a phone number where to call in case of questions.

The intervention also included an interview with the patient two months after discharge in order to review the agreement, adapt treatment if needed and reinforce the intervention. Informative materials are given once again.

Every visit (both at discharge and after two months) took an average of 30-40 minutes each.

Patients assigned to the control group receive the usual care given in each centre.

### Assessment of effects

The main outcome is the proportion of patients in each therapeutic objective (Table 1). These objectives form part of the intervention group's physician/patient agreement. There was direct measurement of blood pressure, blood lipids and anthropometric variables. Medication compliance, as well as smoking status and exercise were self reported by the patient.

**Table 1:** Objectives and indicators

Objectives	Indicators
Giving up smoking	% of patients who gave up smoking % of patients who continued to smoke but reduced tobacco consumption

Having BMI < 25	% of patients with BMI ≥ 25 who reduced it by at least 10% % of patients with BMI ≥ 25 who reduced it by at least 5% % of patients who went from BMI ≥ 25 to BMI < 25 % of patients with BMI ≥ 30 who reduced it by at least 10% % of patients with BMI ≥ 30 who reduced it by at least 5% % of patients who went from BMI ≥ 30 to BMI < 30

Doing regular exercise	% of patients who do physical exercise at least three times a week for at least 20/30 minutes % of patients who do physical exercise at least five times a week for at least 20/30 minutes (only this exercise indicator in diabetic patients)

Controlling lipid levels	% of patients with total cholesterol < 175 mg/dl % of patients with LDL cholesterol < 100 mg/dl (< 70 mg/dl in diabetics) % of patients with LDL cholesterol < 70 mg/dl % of patients with HDL cholesterol ≥ 40 mg/dl % of patients with triglycerides < 150 mg/dl

Controlling hypertension	% of patients with hypertension who have had their blood pressure controlled since discharge % of patients with blood pressure < 135/80 mmHg (< 130/80 mmHg in diabetics)

Taking prescribed medication	% of patients taking AAS (6 months) when prescribed at discharge % of patients taking Clopidogrel (6 months) when prescribed at discharge % of patients taking beta blockers (6 months) when prescribed at discharge % of patients taking ACE-inhibitors (6 months) when prescribed at discharge % of patients taking angiotensin receptor blocking agent Type II (ARA II) (6 months) when prescribed at discharge % of patients taking statins (6 months) when prescribed at discharge

All data, both at baseline and follow-up, were collected directly from the patients (or from hospital records, in the case of biochemistry results) by the participating physicians.

### Plan of analysis

The comparison between intervention and control groups regarding the proportion of patients who achieve each one of the objectives (smoking, arterial hypertension (AHT), cholesterol, obesity) was done by means of the chi-square test. The effect of intervention on variables indicated, adjusting for baseline differences (not expected, taking into account the design chosen) which might be confounding factors was done fitting a logistic regression model for each objective (dependent variable: achievement/non-achievement).

In order to compare average change (basal-final) in continuous variables between intervention and control groups, a model was adjusted by means of analysis of variance (ANOVA) in which the dependent variable is the change occurring between basal visit and final visit, the main independent variable being whether they belonged to the intervention or control groups, and adjustment variables are those which display significant baseline differences between control and intervention groups, among patients who completed the follow-up (analyses not presented): age group, level of education, previous history of stroke, receiving medication for high cholesterol and situation regarding the consumption of tobacco prior to admission.

### Subgroup analysis

In the protocol, pre-specified subgroup analyses by age group (< 70 years of age and ≥70 years of age), gender, and the existence or not of a personal history of ischemic heart diseases were included. These are not presented in this article.

### Calculation of Sample Size

The original calculation was carried out for a minimum of 40 participating centres and 1,200 patients recruited (around half of them in each group). Assuming an achievement rate of 50% in the control group (i.e. proportion of patients in LDL objectives), this sample size would give the study a power of 90% to detect differences of 10% in the achievement of objectives between the two groups for an alpha error of 0.05.

Recruitment of centres was more successful than anticipated and the large number of participating centres and patients recruited has enabled us to improve our ability to detect differences between the groups. The actual number of 1510 subjects improves the power to detect the above mentioned difference to 97,5%. Conversely, for a proportion of 50% in the control group and an alfa error of 0.05, its actual sample size gives the study a power of 90% to detect a minimum difference of 8.3% between the two groups.

## Results

Baseline characteristics of the intervention and control groups are displayed in Table 2. The randomization process produced differences in a few potentially important variables from the total number assessed. Summing up, the intervention group had, on average, a slightly lower age and a greater proportion of smokers at the onset of the acute coronary syndrome, while the proportion of diabetics was smaller. The visit at two months was effectuated as planned by 726 of the 867 patients in the intervention group (84%).

**Table 2:** Comparison of Basal Characteristics of Intervention and Control Groups*

	Intervention (n = 867)	Control (n = 890)	p-value
Males	77.7	75.6	0.301
Age (years old) (mean ± sd)	62.1 ± 11.6	63.6 ± 11.4	0.007
Present diagnosis AMI	65.9	64.6	0.304
Family history of ischemic cardiomyopathy	18.7	17.9	0.657
Certain previous diagnosis of ischemic cardiomyopathy	31.5	29.1	0.276
Personal history:			
Stroke	4.8	6.4	0.156
Peripheral arteriopathy	8.8	9.3	0.683
Renal failure	5.3	5.8	0.624
Arterial hypertension	58.9	60.1	0.616
Hypercholesterolemia	51.4	50.7	0.748
Diabetes	25.3	29.6	0.044
Obesity	31.3	34.2	0.195
Smokers	33.3	26.1	0.001
BMI (kg/m^2^) (average ± sd)	28.2 ± 4.2	28.3 ± 4.3	0.627
BMI ≥ 30 kg/m^2^	29.6	30.4	0.932
Abdominal perimeter (men/women) ≥ 102/88 cm	47.8	51.8	0.172
Abdominal perimeter (men/women) ≥ 94/80 cm	74.0	74.3	0.929

* Results expressed as % unless otherwise indicated

**Figure 1:**
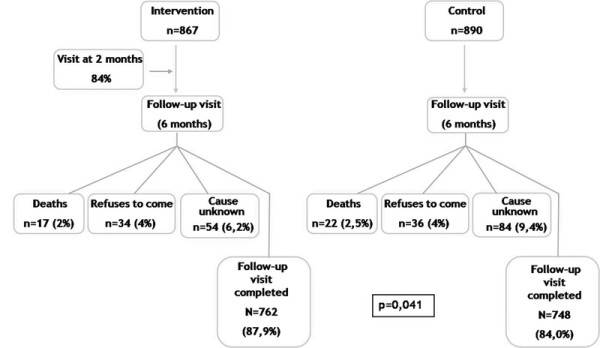
**Flow-chart of follow-up of patients included in trial**.

During the six-month follow-up period, 17 patients died in the intervention group (2%) and 22 in the control group (2.5%), a non-significant difference. Another 88 patients from the intervention group and 120 from the control group failed to realize the follow-up visit. This resulted in a difference in the proportion of patients who completed the follow-up visit (87,9% vs 84,0, p = 0,041), mainly due to "unknown cause" (Figure 1).

The main results of the study are summarized in Table 3, which presents the proportion of patients achieving the various objectives defined and the degree of improvement in the related quantitative variables. It also presents the odds ratios of achieving different objectives, adjusted for those variables which showed differences between the two groups at the beginning of the study (age group, educational level, previous history of stroke, medication for cholesterol and tobacco consumption prior to admission). As achieving treatment objectives is something desirable, odds ratio above 1 are good in this context. For continuous variables, instead of adjusted odds ratios, the table presents adjusted differences (for the same variables) in the mean changes between the two groups.

**Table 3:** Degree of Achievement of Objectives at Six Months in Both Groups

Objectives (nI/nC)	Intervention (n = 762)	Control (n = 748)	Adjusted^γ^ Odds ratio	Adjusted OR 95% CI	p-value
Given up smoking (253/200)	76.3	71.0	1.25	0.81-1.94	0.309
BMI ≥ 25 kg/m^2 ^(567/547)					
< 25 kg/m^2^	9.9	7.8	1.21	0.79-1.86	0. 373
⇓ BMI ≥ 10%	7.4	6.8	1.15	0.72-1.84	0.564
⇓ BMI ≥ 5%	25.0	19.7	1.31	0.98-1.76	0.068
BMI ≥ 30 kg/m^2 ^(212/209)					
< 30 kg/m^2^	25.9	24.9	1.04	0.66-1.63	0.860
⇓ BMI ≥ 10%	11.3	10.5	1.15	0.61-2.17	0.666
⇓ BMI ≥ 5%	32.1	27.8	1.26	0.82-1.93	0.300
Changes in BMI (average) (kg/m^2^)*	- 0.5	- 0.2	0.35*	0.16-0.55*	< 0.001
Changes in abdominal perimeter (average) (cm)*	- 1.6	- 0.6	1.03*	0.00-2.06*	0.05
Exercise 5 times/week	29.4	23.4	1.29	1.02-1.64	0.033
Exercise 3 times/week	48.4	41.0	1.29	1.05-1.59	0.017
Cholesterol < 175 md/dl	64.7	56.5	1.46	1.17-1.81	0.001
LDL < 100 mg/dl (70 in diabetic patients)	47.9	44.8	1.09	0.87-1.35	0.449
BP < 135/80 mmHg (130/80 in diabetic patients)	46.7	42.4	1.14	0.91-1.41	0.253
Taking of medication (percentage who take it on final visit, out of total given medication on discharge from hospital)					
Aspirin (712/698)	97.5	96.0	1.54	0.84-2.83	0.161
Clopidogrel (571/533)	72.9	74.3	0.91	0.69-1.20	0.499
Beta-blockers (621/590)	94.4	94.6	0.93	0.56-1.54	0.769
ACE-inhibitors (400/387)	86.8	84.0	1.31	0.87-1.98	0.192
Angiotensin receptor blocking agent Type II (ARA II) (86/97)	84.9	86.6	0.83	0.35-1.95	0.664
Statins (677/635)	98.1	95.9	0.83	0.35-1.95	0.029

Results in intervention and control columns expressed as % of total number of patients affected by this objective as indicated in the first column, unless otherwise indicated

^γ ^Differences in means (ANOVA model) or odds ratios [OR, logistic regression] adjusted for age group, education level, history of stroke, medication for cholesterol and tobacco consumption prior to admission, variables with statistical significant differences at baseline in the group of patients included in this analysis. Reference group for all estimates: control group). See interpretation of OR in the results section.

* In these continuous outcome variables, (changes in body mass index [BMI] and abdominal perimeter) the coefficients of the ANOVA model adjusted for the same variables, instead of odds ratios, are presented.

nI/nC: Number of patients in intervention/control groups on which the percentages presented are based when they are not the same as the total indicated in the corresponding column.

CI: Confidence interval

To sum up, the tendency is always towards a greater number of people achieving objectives within the intervention group, but differences are only statistically significant in the case of mean differences in body mass index, waist measurement, patient's own declaration of exercise realized and total cholesterol level. With regard to body mass index, even though the mean of this variable is lower in the intervention group, the proportion of patients achieving the predetermined objectives is small, both among those who are overweight and those who are obese, and there are no significant differences between the intervention and control groups, even in the case of the less ambitious objectives (reduction of at least 5% of initial body mass index).

## Discussion

This trial carries out a short-term assessment of a simple intervention in the relationship between physician and patient in which its main element is the negotiation between the two and the ability to reach agreements regarding which steps to take in relation to care and the objectives to be fulfilled. The findings can be summed up as a moderate but potentially important effect on variables relating to lifestyle (physical exercise and body mass index), which are usually the most difficult aspects to modify. It also demonstrates that the vast majority of patients state that they take the medication and that an intervention such as that described above contributes nothing in this respect, at least as far as we can judge from the statements of the patients themselves, with all the limitations that this implies.

Although many studies have attempted to improve compliance levels in activities in secondary prevention after an acute coronary syndrome, firstly, many of these are focused exclusively on the moment of hospital discharge, and secondly, the intervention takes place only on the physician [14-17]. Thus, as a new element, our study prolongs the time of the intervention beyond hospital discharge, and moreover includes the patient as a participant in the achievement of objectives. The agreement signed by the patient, and early monitoring in the clinic (two months after discharge) to reinforce the message, were found to be related to a reduction in BMI, in exercise carried out continuously and in the objective of reaching levels of total cholesterol. These results suggest that this type of interventions can result in an improvement on the quality of care in this type of patients.

### Intervention type and results

Early monitoring of coronary patients after discharge subsequent to a coronary event has been related, in various studies, both with higher rates of pharmacological compliance and with a favourable impact on patients' evolution, with a reduction in hospitalizations and mortality. Guidelines also recommend early monitoring (2 to 6 weeks) even in low-risk patients, after an acute coronary syndrome [19,20]. In our study, the intervention performed is compared with common practice. This is variable among centres, but common practice is to refer a high proportion of patients to the general practitioner on hospital discharge. In-depth interviews between cardiologist and patient prior to discharge are uncommon, due to the progressive reduction in time spent in hospital by these patients. Our study, unlike others, failed to demonstrate an improvement in levels of pharmacological compliance. In part, this could be explained by the absence of a control group in these studies, so that the benefit reflected in these studies may always be overrated. The positive effect of an early visit, that took place after two months in our study, is related to the fact that hospital discharge is usually a critical moment where the change between hospital and out-patient care (whether primary or specialized care) is a period of vulnerability, when high rates of errors related to medication and incomplete or inappropriate information are present [21,22]. However, an early follow-up visit can help prevent problems of primary compliance, which in post-acute myocardial infarct patients can be as high as 17.7% in cardiology medication [23].

Another tool used in our study is an agreement signed by both physician and patient on the objectives to be achieved during follow-up. This strategy is an attempt to involve the patient as jointly responsible for his/her care and avoid the relinquishment of his own function within the health service which cares for him. This is justified by the fact that the patient's collaboration and responsibility are fundamental in aspects such as long-term modification of lifestyle. Our study demonstrates that this tool can be useful in achieving modifications in lifestyle. However, though we consider that it is a key element in the intervention, our study does not allow us to evaluate the different components of the intervention independently.

In our study we carried out an analysis, after 6 months, of whether the intervention influenced patients' self-reported estimation of their pharmacological compliance. We found no difference between the two groups at six months except in the percentage of use of statins, with a loss of only 1.9% in the intervention group, as against 3.9% in the control group. The absence of this effect could be explained by the short follow-up period involved (6 months), especially because the time that elapses since the acute event is recognized as a factor contributing to non-compliance with treatment. Despite a high self-reported adherence to the prescribed medication, there is still a low proportion of patients achieving clinical goals. A similar observation has been reported among coronary heart disease patients in a recent survey in eight European countries [24], and in other studies where achievement of lipid lowering goals may be as low as 30% among high risk coronary heart disease patients [25].

In summary, it is important to point out that the improvements of the intervention concentrated in hygienic-dietetic measures (regular practice of exercise and weight loss and a non-significant tendency in the reduction in smoking) variables traditionally with lower compliance and greater difficulties for improvement than the pharmacological ones in other cardiovascular conditions (i.e. heart failure) [26,27]. It is easier for a patient to take medication than to change his lifestyle. Our study suggests that such changes are possible, at least in the short term, by means of a simple and feasible intervention that includes a component to increase the compromise from the patient (patient-physician agreement) and a visit to reinforce the messages. Nevertheless, the results also show that performance is still suboptimal, and that there is a need of additional improvements.

## Limitations

Compliance rates are typically higher among patients with acute conditions compared with those suffering from chronic pathologies, especially from 6 months on [28,29]. This could be a limitation to the scope of our study, given that it is a post-acute coronary syndrome study and only covers monitoring up to 6 months. An additional potential limitation is that physical activity is declared by the patient him/herself, and there are no mechanisms within the study that could make this more objective. This is the usual practice in a clinical environment, but in this study poses the doubt that the differences might be due to a few patients with a contract not wanting to admit breaking it. This does not seem to be the reason because the patient-physician agreement includes other self-reported variables besides lifestyle (i.e medication adherence) where the effect is not seen.

On the other hand, it has been affirmed that collaborative follow-up protocols which actively involve primary care can be more effective than those performed by one professional on his/her own involved in care of the patient. The design of our study did not include involvement of the primary care physician, a fact which could limit the generalization of our results [30,31].

## Conclusion

Taking medication is easier for a patient than changing his/her lifestyle. As a conclusion and as possible summary message with clinical application, our study suggests that, at least in the short term, lifestyle changes are achievable by intensifying the responsibility of the patient himself.

## Competing interests

The authors declare that they have no competing interests.

## Authors' contributions

JM conceived of the study and participated in its design, acquisition of funding, coordination and statistical analysis, and drafted the manuscript. JJGD participated in the design of the study, recruitment and follow-up of patients and drafted the manuscript. MISP performed the statistical analysis of data and revised critically the manuscript. ILG participated in the design of the study, recruitment and follow-up of patients and revised critically the manuscript.

NME participated in the design of the study, recruitment and follow-up of patients and revised critically the manuscript. EdTG participated in the design of the study and revised critically the manuscript. JMCF participated in the design of the study and revised critically the manuscript. ACB conceived of the study and participated in its design, acquisition of funding and coordination, and revised critically the manuscript. All authors read and approved the final manuscript.
